# Interferon-stimulated gene 15 in hepatitis B-related liver diseases

**DOI:** 10.18632/oncotarget.11955

**Published:** 2016-09-10

**Authors:** Nghiem Xuan Hoan, Hoang Van Tong, Dao Phuong Giang, Nguyen Linh Toan, Christian G. Meyer, C.-Thomas Bock, Peter G. Kremsner, Le Huu Song, Thirumalaisamy P. Velavan

**Affiliations:** ^1^ Institute of Tropical Medicine, University of Tübingen, Tübingen, Germany; ^2^ 108 Military Central Hospital, Hanoi, Vietnam; ^3^ Vietnamese-German Center for Medical Research, Hanoi, Vietnam; ^4^ Department of Pathophysiology, Vietnam Military Medical University, Hanoi, Vietnam; ^5^ Department of Infectious Diseases, Robert Koch Institute, Berlin, Germany

**Keywords:** HBV infection, liver diseases, ISG15, *ISG15* polymorphism, ISGlation, Pathology Section

## Abstract

This study investigates the association of Interferon-stimulated gene 15 (*ISG15*) polymorphisms, ISG15 serum levels and expression with HBV-related liver diseases. The *ISG15* promoter and the two exons of the gene were screened for polymorphisms in 766 HBV-infected patients and in 223 controls. Soluble ISG15 levels were measured by ELISA. *ISG15* mRNA expression was quantified by qRT-PCR in 36 tumor and adjacent non-tumor tissues. The exon 2 allele *rs1921A* was found associated with decreased progression of HBV-related liver diseases (LC *vs.* CHB: OR = 0.6, 95%CI = 0.4-0.8, adjusted *P* = 0.003; HCC *vs.* CHB: OR = 0.6, 95%CI = 0.4-0.9, adjusted *P* = 0.005). The *rs1921AA* genotype was associated with low levels of AST, ALT and total bilirubin, but with high prothrombin levels (*P* < 0.05). ISG15 serum levels were higher among HBV patients compared to controls (*P* < 0.0001) and positively associated with HBV-related liver diseases, with highest levels among LC patients. ISG15 levels were correlated with HBV-DNA loads (*P* = 0.001). In non-tumor tissues from HCC patients, *ISG15* mRNA expression was increased in HBV compared to non-HBV infection (*P* = 0.016). The *ISG15* rs1921 variant and *ISG15* expression are associated with HBV-related liver diseases. Taken together, ISG15 appears to be a proviral factor involved in HBV replication and triggering progression of HBV-related liver diseases.

## INTRODUCTION

Although effective vaccines for hepatitis B virus (HBV) infections are available, HBV-related liver diseases remain a health problem of considerable concern with 250 million chronic carriers globally [1]. In Vietnam, the prevalence of HBV infection currently ranges from 10% to 20% in general population and HBV-related liver diseases are foreseen and predicted to be a notable public health burden in the next decades [2]. HBV causes various forms of infection, ranging from an asymptomatic carrier status to liver cirrhosis (LC) and life-threatening hepatocellular carcinoma (HCC) [1]. Important factors influencing disease progression include the patient's age, duration of HBV infection, and host-virus interactions. HBV has evolved various mechanisms to evade both innate and adaptive immune responses in order to establish persistent infections [3].

Currently, nucleos(t)ide analogues and Peg-Interferon alpha are the main classes of antivirals to treat chronic hepatitis B (CHB) [4,5]. IFN-α mediates signal transduction by binding to its receptors. Tyrosine phosphorylation of signal transducer and activator of transcription 1 (STAT1) and STAT2 leads to the formation of transcriptional complexes, which translocate to the nucleus and activate expression of certain genes [6]. IFN-stimulated gene factor 3 (ISGF3) is composed of phosphorylated forms of STAT1, STAT2 and interferon regulatory factor 9 (IRF9). This complex binds to interferon (IFN)-stimulated response elements (ISREs) located in the promoters of interferon-stimulated genes (ISGs) [6]. Among ISGs, the human ISG15 is a 15 kDa protein encoded by *ISG15* located on chromosome 1p36.33 (OMIM# 147571) [7]. ISG15 is a strongly induced protein in various cellular processes [8,9]. It exists in free and/or conjugated forms (ISGlation), covalently conjugated to protein targets via consecutive action of conjugating enzymes such as ubiquitin activating E1 (UbE1L), E2-conjugating enzyme (UbcH8) and E3 ligases. The ubiquitin-specific protease 18 (USP18), which has deconjugating protease functions, cleaves ISG15′s substrates and removes ISG15 (deISGylation) from ISG15 conjugates [8].

Several studies have indicated that aberration of cell signaling in the ISG15 pathway perturbates ISG15 regulation and causes malignant transformation of various human cancers [10-14]. ISG15 overexpression in liver tumor tissue is associated with pathology and poor outcome of HCC patients [12,13]. ISG15 plays also a role in the response to many viral infections [9,15-18]. A recent study has shown that knockdown of *ISG15* results in suppression of hepatitis C virus (HCV) replication *in vitro* by promoting the IFN response, suggesting involvement of ISG15 in regulating HCV replication [19,20]. Furthermore, patients who respond favorably to IFN treatment of hepatitis C have low expression levels of ISG15 and its conjugates in the liver compared to non-responders [21,22]. So far, two studies using mouse models have documented the role of ISG15 in HBV pathogenesis. They concluded that mice injected with murine IFN-α expression plasmid along with HBV had higher expression levels of ISG15 [23], and an ISG15-deconjugating enzyme (USP18) reduced expression is associated with rapid HBV clearance [24]. However, the functional role of ISG15 in the HBV replication cycle, immune response and clinical progression of HBV-related liver diseases remains poorly understood. We investigated possible *ISG15* associations, of ISG15 serum levels and *ISG15* expression with outcomes of HBV infection and progression of HBV-related diseases.

## RESULTS

### Study participants

Baseline characteristics of the clinically well-characterized 766 HBV patients and of the 223 healthy controls (HCs) are given in Table 1. The median age of patients increased according to the progression of disease (*P* < 0.001). ALT, AST, total bilirubin and direct bilirubin levels as well as HBV loads were higher among CHB patients compared to other subgroups (*P <* 0.0001). As expected, albumin and prothrombin levels and platelet counts were lower in LC patients compared to the other patient groups (*P* < 0.0001). AFP levels were higher in HCC patients compared to the CHB and LC subgroups (*P* < 0.0001). Of the 36 HCC patients who underwent surgery, 32 were males and most patients (27/36, 75%) were aged between 40-60 years. According to Barcelona Clinic Liver Cancer (BCLC) staging [25], 25/36 (69%) HCC patients were in stage A and 11/36 (31%) HCC patients in stage B. Among all HCC patients, 17/36 (47%) had HBV infection, 2/36 (6%) had HCV infection, and 17/36 (47%) showed non-HBV/HCV-related HCC (Suppl. Table 1).

**Table 1:** Characteristics HBV patients segregated according to clinical presentation and healthy controls

Characteristics	CHB (*n*= 262)	LC (*n*= 241)	HCC (*n*= 263)	HC (*n*= 223)
Age (years)	42 [18-82]	54 [18-84]	58 [18-81]	37 [18-69]
Male (%)	74.5	83.7	92.2	66.4
AST (IU/L)	49 [14-7700] ^‡^ β	75 [15-1221]	60 [17-2158]	<40
ALT (IU/L)	58 [9-4908] ^‡^ β	56 [8-1426]	46 [11-832]	<40
Total bilirubin (mg/dL)	16 [6-357] ^‡^ β	30 [3-752]	18 [6-419]	<17
Direct bilirubin (mg/dL)	5 [1-226] ^‡^ β	12 [1-450]	6.5 [1.2-214]	<5
Albumin (g/L)	41 [23-50]	33 [20-47] ^‡^β	38 [22-49]	>35
Prothrombin (% of standard)	87 [30-180]	54 [15-101] ^‡^β	80 [20-149]	>70
WBC (x10^3^/mL)	6 [3.6-13.9]	5.6 [1.7-20.5)	6 [6.6-16]	4 - 10
RBC(x10^6^/mL)	4.8 [3.2-6.8)	3.9 [1.9-6.7)	4.5 [2.1-6.8)	4 - 9
PLT (x10^3^/mL)	195 [19-472]	89 [18-441] ^‡^ β	166 [34-389]	150-300
HBV-DNA (copies/mL)	5.8×10^5^ [2×10^2^- 8.4×10^10^] ^‡^ β	1.8×10^4^ [1.8×10^2^- 4.7×10^9^]	8.2×10^5^[10^2^-3×10^10^]	NA
AFP (IU/L)	2.9 [1.5-320]	36 [1.2-400]	196 [1.1-480] ^‡^ β	<5

CHB, chronic hepatitis B; LC, liver cirrhosis; HCC, hepatocellular carcinoma; HC, healthy control; RBC, red blood cells; WBC, white blood cells; PLT, platelets. AST and ALT, aspartate and alanine amino transferase; AFP, alpha-fetoprotein; IU, international unit; NR, normal range, NA, not applicable. Values given are medians and ranges. (β) Kruskal-Wallis test was used to test differences of nonparametric data.

‡ *P* < 0.0001 for comparisons with other groups.

### Association of *ISG15* rs1921 variant with HBV-related liver diseases

The genotype frequencies of the *ISG15* rs1921 variant in HCs were in Hardy-Weinberg equilibrium (*P* = 0.166), whereas other promoter and exonic variants were not. Therefore, only *ISG15* rs1921 was considered for further analyses. The genotype and allele frequencies in different subgroups and the association analyses are presented in Table 2. Genotype and allele frequencies of *ISG15* rs1921 did not differ between HBV patients and controls, indicating that *ISG15* rs1921 is not associated with HBV infection *per se*. However, the *rs1921GA* genotype occurred more frequently among CHB patients compared to the LC and HCC subgroups (LC vs. CHB, OR = 0.5, 95%CI = 0.3-0.8, adjusted *P* = 0.036; HCC *vs.* CHB, OR = 0.5, 95%CI = 0.3-0.8, adjusted *P* = 0.014). The minor allele *rs1921A* was more frequent in CHB than in HCC and LC patients (LC *vs.* CHB, OR = 0.6, 95%CI = 0.4-0.8, adjusted *P* = 0.003; HCC *vs.* CHB, OR = 0.6, 95%CI = 0.4-0.9, adjusted *P* = 0.005). In a dominant genetic model, we also observed that minor allele *rs1921A* was associated with an increased protection against LC and HCC (LC *vs.* CHB, OR = 0.5, 95%CI = 0.3-0.7, adjusted *P* = 0.0016; HCC *vs.* CHB, OR = 0.5, 95%CI = 0.4-0.8, adjusted *P* = 0.003) (Table 2). These results indicate that *rs1921A* may contribute to a decreased risk of progression to LC and HCC in HBV infection.

**Figure 1:**
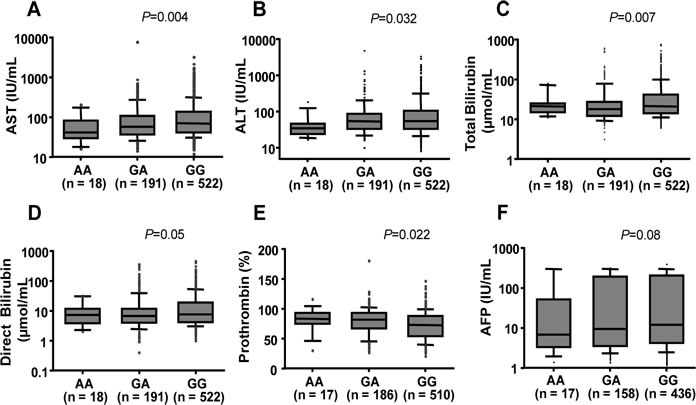
Association between *ISG15* variant with clinical parameters in HBV patients Box-plots illustrate median values with 25 and 75 percentiles with whiskers to 10 and 90 percentiles; *P* values were calculated by Kruskal-Wallis tests. AST and ALT, aspartate and alanine amino transferase; AFP, alpha-fetoprotein.

In order to explore the influence of *ISG15* rs1921 on the clinical outcome of HBV-related liver disease, we compared clinical parameters among patients with different *ISG15* rs1921 genotypes (*GG*, *GA* and *AA*). Pathological liver function tests as indicated by high levels of AST, ALT, total bilirubin, direct bilirubin and by low prothrombin levels were observed rather in HBV patients with the *rs1921GG* genotype (*P* < 0.05) (Figure 1) than in the other subgroups. AFP levels and viral loads were higher in patients with *rs1921GG* compared to those with either *rs1921AA* or *rs1921GA*. However, the difference was not significant (*P*>0.05) (Figure 1 and Suppl. Figure 1A).

**Table 2:** Association of *ISG15* variant (rs1921) with HBV-related liver diseases

*ISG15* variant	CHB *n*(%)	LC n(%)	HCC *n*(%)	HC *n*(%)	HBV patients vs. HCs		LC vs. CHB		HCC vs. CHB		HCC vs. LC	
**(rs1921)**	***n*= 262**	***n*= 241**	***n*= 263**	***n*= 223**	**OR (95%CI)**	***P***	**OR (95%CI)**	***P***	**OR (95%CI)**	**P**	**OR (95%CI)**	***P***
Codominant												
*GG*	161(61.5)	180(74.7)	198(75.3)	158(70.7)	Reference		Reference		Reference		Reference	
*GA*	92(35.1)	57(23.7)	58(22.1)	56(25.2)	1.2(0.8-1.6)	0.43	**0.5 (0.3-0.8)**	**0.006**	**0.5 (0.3-0.8)**	**0.014**	0.9(0.6-1.4)	0.53
*AA*	9(3.4)	4(1.7)	7(2.6)	9(4.1)	0.7 (0.3-1.6)	0.89	0.4 (0.1-1.5)	0.17	0.3 (0.1-1.2)	0.12	1.9(0.5-6.7)	0.47
Allele												
*G*	414(79)	417(86.5)	454(86.3)	416(84.9)	Reference		Reference		Reference		Reference	
A	110(21)	65(13.5)	72(13.7)	74(15.1)	1.1(0.8-1.4)	0.90	**0.6 (0.4-0.8)**	**0.003**	**0.6 (0.4-0.9)**	**0.005**	1.0(0.7-1.5)	0.95
Dominant												
*GG*	161(61.5)	180(74.7)	198(75.3)	157(70.7)	Reference		Reference		Reference		Reference	
*GA&AA*	101(38.5)	61 (25.3)	65 (24.7)	65(29.3)	1.1(0.8-1.5)	0.62	**0.5 (0.3-0.7)**	**0.0016**	**0.5 (0.3-0.8)**	**0.003**	0.9(0.6-1.4)	0.83
Recessive												
*GG&GA*	253(96.6)	237 (98.3)	256(97.4)	213(95.9)	Reference		Reference		Reference		Reference	
*AA*	9(3.4)	4(1.7)	7(2.6)	9(4.1)	0.7(0.3-1.5)	0.34	0.5 (0.1-1.8)	0.25	0.6 (0.2-1.9)	0.32	1.9(0.5-68)	0.31

CHB, chronic hepatitis B; LC, liver cirrhosis; HCC, hepatocellular carcinoma; HC, healthy controls; n = numbers genotyped; OR, Odd Ratio. *P* values were calculated using binary logistic regression model adjusted for age and gender. Bold values reflect statistical significance.

### ISG15 serum levels and HBV-related liver diseases

**Figure 2:**
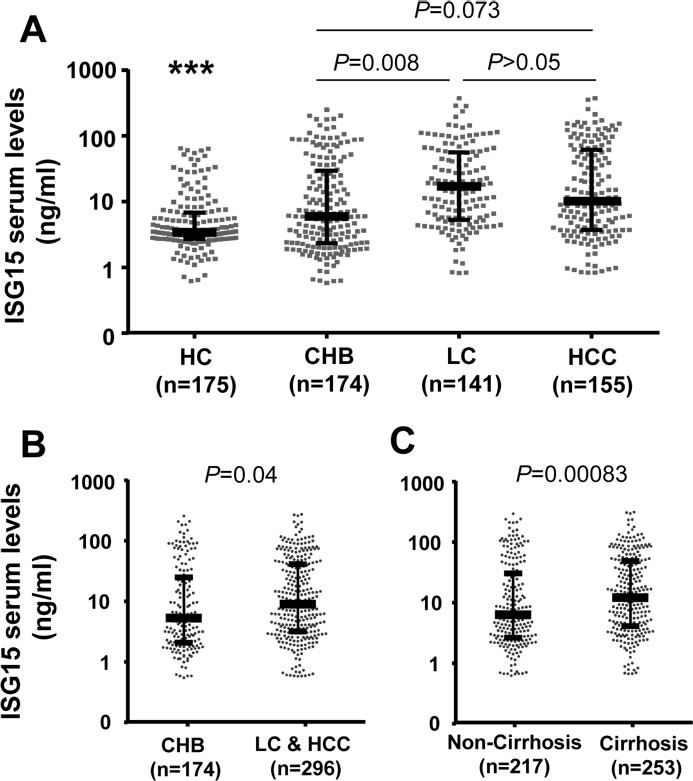
ISG15 serum levels in healthy individuals and in HBV patient sub-groups HC, healthy controls; CHB, chronic hepatitis B; LC, liver cirrhosis; HCC, hepatocellular carcinoma; Cirrhosis: combination of patients with LC and patients with both HCC and LC; non-cirrhosis: combination of CHB patients and HCC patients without liver cirrhosis. (***): *P* < 0.0001 for comparison with other groups. Dot plots illustrate medians with inter-quartile range. *P* values were calculated by Mann-Whitney-Wilcoxon test.

We quantified ISG15 levels in serum samples of 470 HBV patients and 175 healthy controls. ISG15 serum levels were significantly lower in the control group (median: 3.4 ng/ml) compared to all HBV patients (median: 8.1 ng/ml), and compared to the median values of the different subgroups (CHB, 6.5 ng/ml; LC, 12 ng/ml; HCC 8.0 ng/ml; *P* < 0.0001) (Figure 2A). Among the HBV patients, ISG15 levels were lower in the CHB than in the combined LC and HCC subgroups (*P* = 0.008 and 0.04; respectively) (Figure 2A, 2B). In addition, all LC, including HCC patients with concomitant LC, had significantly higher ISG15 levels than non-LC patients (*P* = 0.00083) (Figure 2C). However, ISG15 levels did not differ significantly between patients with and without HCC (HCC vs. CHB+LC: *P*>0.05) (Figure 2A and Suppl. Figure 2). These results show that ISG15 induced by HBV infection may play a role in progression of HBV-related liver diseases.

### *ISG15* rs1921 variant and ISG15 serum levels

We analyzed the association of the *ISG15* rs1921 variant with ISG15 serum levels in HBV patients and controls. ISG15 serum levels in HBV patients with the genotype *rs1921GG* were marginally higher than those in HBV patients with either *rs1921AA* or *rs1921GA* genotypes (*P* = 0.089) (Suppl. Figure 1B). Among controls, ISG15 levels did not differ among individuals with the various genotypes (*P*>0.05).

### ISG15 serum levels and viral loads

**Figure 3:**
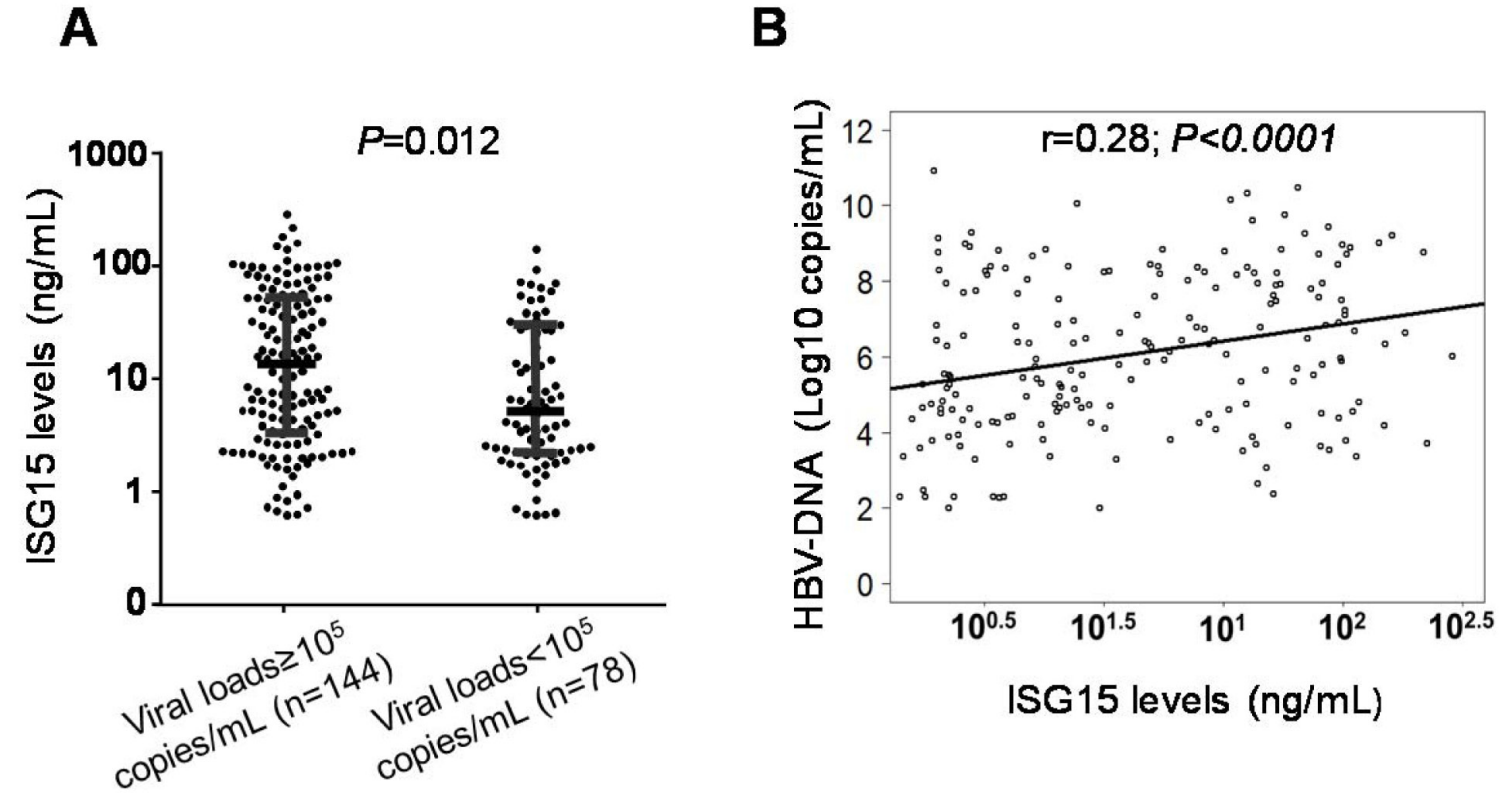
Association of ISG15 serum levels with viral loads in HBV patients **A.** Dot plots illustrate median values with 25 and 75 percentiles with whiskers to 10 and 90 percentiles. *P* values were calculated by Mann-Whitney-Wilcoxon test. **B.** Linear regression analysis indicates a positive correlation between ISG15 serum levels and HBV-DNA in CHB patients. Non parametric Spearman's rank correlation coefficient (r) and *P* values are presented.

Of 766 HBV patients, 222 were available for analyses of the correlation between ISG15 serum levels and HBV-DNA viral loads. ISG15 serum levels were significantly higher in patients with high viral loads (viral loads ≥10^5^ copies/ml) compared to patients with lower levels (viral loads < 10^5^ copies/ml) (Figure 3A). In a simple linear regression analysis, the ISG15 serum levels were positively correlated with HBV-DNA loads (r = 0.28, *P* < 0.0001) (Figure 3B). High levels of HBV replication and LC were, in a multivariate linear regression model, independently associated with increased ISG15 serum levels (*P* = 0.0011 and 0.008, respectively) (Table 3).

**Table 3:** Factors associated with ISG15 serum levels in HBV patients

Variables	Univariate analysis	Multivariate analysis	
	*P* value	*P* value	Coefficient β
Age	0.31	0.57	−0.0096
Gender (male vs. female)	0.4	0.3	0.13
HBV-DNA (log10 copies/mL)	**<0.0001**	**<0.0001**	0.14
ALT (IU/L)	0.42	0.57	−0.00013
AST (IU/L)	0.46	0.88	−0.00003
Albumin (g/L)	0.30	0.12	0.02
Total Bilirubin (μmol/L)	0.13	0.8	−0.00017
Prothrombin (% of standard)	**0.0015**	0.16	−0.0004
Platelets (x10^3^/mL)	0.47	0.11	−0.0011
HCC vs. non-HCC	0.9	0.12	0.2
Cirrhosis vs. non-Cirrhosis	**0.00032**	**0.009**	0.3
ISG15 genotype (GA vs. AA)	0.55	0.77	−0.66
ISG15 genotype (GG vs. AA)	0.51	0.62	−1.08

Univariate analysis and multivariate linear regression model for independent factors to correlate ISG15 serum levels with clinical parameters.

### *ISG15* mRNA expression in primary HCC

**Figure 4:**
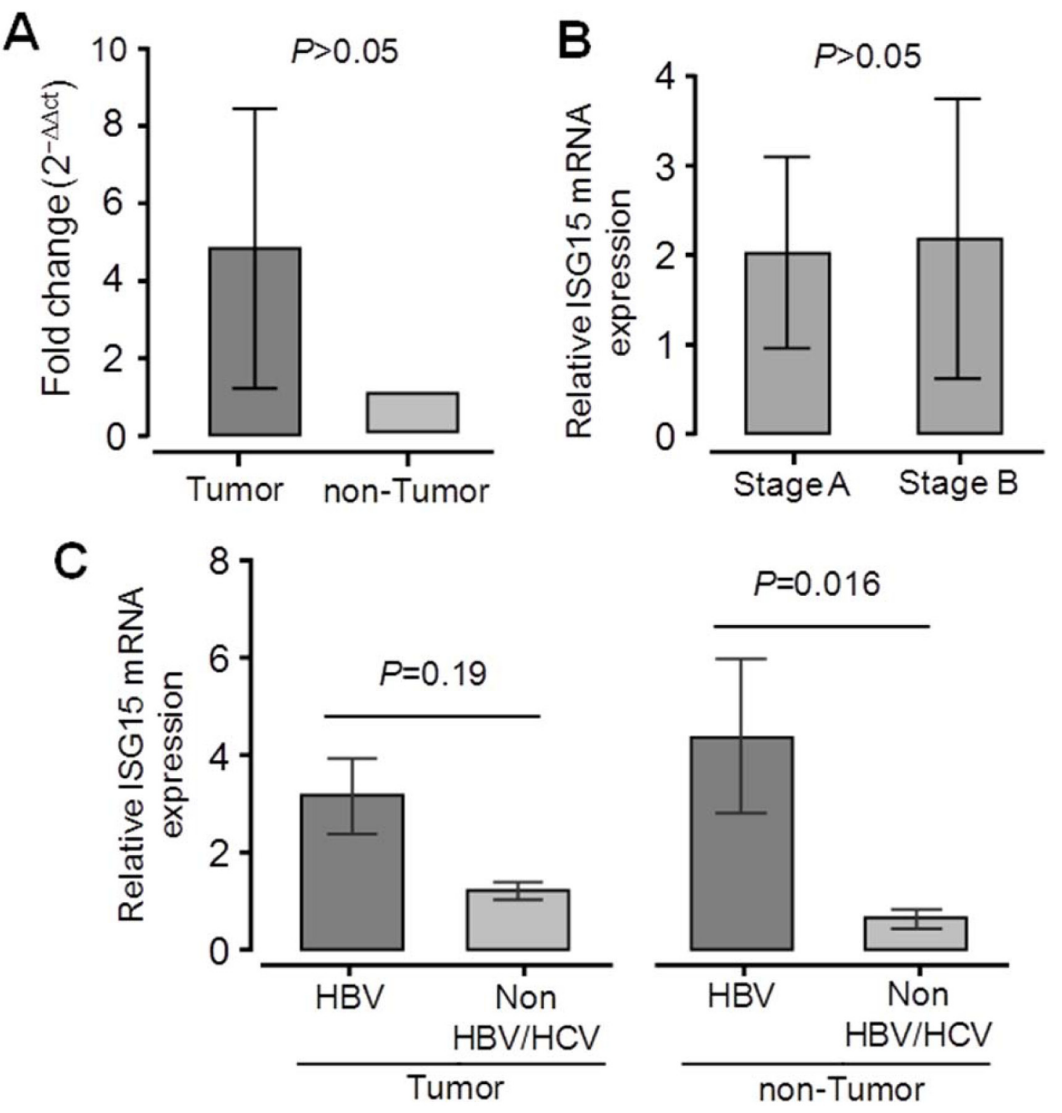
Expression of *ISG15* mRNA in liver specimens from 36 primary HCC patients Relative quantitative real-time PCR (qRT-PCR) analysis of *ISG15* mRNA levels **A.** Relative *ISG15* mRNA expression in tumor and adjacent non-tumor tissues. **B.** Relative *ISG15* mRNA expression in tumor tissues from HCC stage A patients and HCC stage B patients according to the BCLC staging classification. **C.** Relative *ISG15* mRNA expression in liver tissues from patients positive for HBV and in patients negative for both HBV and HCV. Data are shown as mean values with 95% confident intervals. *P* values are calculated by Mann-Whitney-Wilcoxon tests.

We also analyzed expression of *ISG15* mRNA in HCC tissue specimens and in adjacent non-tumor liver tissues retrieved from 36 primary HCC patients. The mean of *ISG15* mRNA expression in liver tumor tissues was higher than in adjacent non-tumor tissues (not significant; Figure 4A). We examined whether *ISG15* mRNA expression can be up-regulated during HCC development, however, *ISG15* mRNA expression did not differ between stage A and stage B HCC tissues (Figure 4B). We further examined whether *ISG15* mRNA expression is associated with HBV infection. *ISG15* mRNA expression was significantly higher in HBV-related non-tumor tissues compared to non-HBV-related non-tumor tissues (*P* = 0.016). A similar trend was observed when comparing HBV-related and non-HBV-related tumor tissues, but the difference was not significant (Figure 4C). These results indicate that increased expression of *ISG15* in liver tissues is regulated by HBV infection.

## DISCUSSION

The interferon signaling pathway constitutes the first-line defense against viral infections. ISGs can regulate the host immune response, which in turn may inhibit viral replication [8,9,26,27]. The role of ISG15 in host defense against invading viral pathogens has previously been documented [9,19,26,27]. ISG15 and its conjugates exhibit both antitumor and oncogenic properties [8,28]. We have investigated the role of *ISG15* variants and ISG15 expression in the progression of HBV-related liver diseases and could show that the non-synonymous *ISG15* rs1921 variant, ISG15 levels and *ISG15* expression are associated with HBV-related liver diseases.

Host genetic factors contribute to the progression of HBV-related liver diseases [29]. To the best of our knowledge, this is the first study showing an association of the *ISG15* rs1921 variant with the clinical outcome of HBV-related liver diseases. The frequency of the *rs1921G* allele in different Vietnamese HBV patient subgroups and controls fits in the range estimated for East Asian of the 1000 Genomes project. The *rs1921GG* genotype and *rs1921G* allele are associated with progression of liver disease and contributes to poor laboratory parameters. The *ISG15* rs1921 exon 2 variant harbors a missense mutation (S355N) and, thus, may influence gene expression and protein modification. ISG15 targets many cellular proteins, including Janus kinase 1 (JAK1), STAT1 and ISGs via ISGlation [30], a process that can regulate HBV pathogenesis. However, our study failed to detect a significant association of *ISG15* rs1921 with ISG15 expression. Our result is similar to a recent observation showing that rs1921 variant had no influence on ISG15 expression in European human immunodeficiency virus (HIV)-1 patients and healthy individuals [31]. It may be more likely that this missense variant may alter the binding affinity to its conjugate. Polyphen and Sift algorithms used for predicting functional consequences of nucleotide substitutions classify this variant as “benign” and “tolerated”, respectively. The association may be due to linkage disequilibrium with another variant (a hitch-hiking effect). However, the effect of rs1921 on ISG15 function and the ISGlation process requires further clarification.

ISG15 and its ISGlation form mediate innate immune responses through IFNs, lipopolysaccharide and double-stranded RNA (dsRNA) stimulation [30]. Higher ISG15 serum levels in HBV patients than in healthy individuals and an increased *ISG15* mRNA expression observed in HBV-related liver tissues compared to non-HBV/HCV-related liver tissues demonstrate that HBV infection induces *ISG15* expression. This is in agreement with the fact that the major host response to viral infection is the production of IFNs, which in turn stimulate *ISG15* expression. The notion of antiviral activities of both ISG15 and ISGlation came from studies using an *ISG15* knockout mice model [15,32]. Mice lacking ISG15 expression were more susceptible to influenza, sindbis and herpes simplex viruses [15]. Recent studies have explored the biological functions of ISG15 and related conjugates that can impair viral replication *in vivo* [18,32-34]. In contrast, our study showed that ISG15 levels were positively correlated with viral loads, implying a contradictory effect of ISG15 on antiviral activities. This is consistent with studies showing that ISG15 can promote HCV replication [19,35,36].

Host immune factors are essential in the immune-pathogenesis of HBV infection through genetic and epigenetic modifications [37,38] and via the effects of cytokines [39]. An ineffective immune response against HBV may result in persistent virus replication and liver inflammation, leading to CHB, LC and HCC [39]. ISG15 appears to act as an immune-modulator regulating the expression of cytokines, in particular of the IFN signalling pathway. Previous studies have indicated that *ISG15* upregulation leads to a blunted immune response to IFN signalling and contributes to a poor outcome of IFN-based therapy in HCV patients [19,40]. In addition, higher levels of ISG15 were observed in treatment failure compared to responders to IFN-based treatment [36,40]. Therefore, high levels of ISG15, combined with clinical, biochemical and histological analyses may be useful to predict the outcome of HBV-related liver disease and may help to identify HBV-infected individuals positively responding to IFN treatment.

The biological function of ISG15 promoting or suppressing tumor growth remains controversial [28] and antagonistic roles of ISG15 in tumorigenesis have been reported [28,41,42]. Several studies have demonstrated that ISG15 is an oncoprotein, as *ISG15* gene expression and its protein conjugates were found elevated in tumor cell lines and in various human malignancies [10,12-14,41,43-46]. High levels of ISG15 serum protein in HCC patients and mRNA expression in liver tumor tissues also suggest that ISG15 may serve as a protumor factor. In contrast, other studies reported that free ISG15 has the potential to induce antitumor responses [47,48]. These discrepancies could be due to the functional differences of free ISG15 and ISG15 conjugates [28,42]. So far it is clear that ISG15 overexpression is crucial in modulating cell growth and in the progression of breast cancer [10,41]. The functional role of ISG15 in HCC, however, is still unclear. ISG15 overexpression is associated with poor clinical outcomes [12,13]. Moreover, knocking down ISG15 by shRNA ISG15 can lead to a remarkable reduction of HCC cell proliferation and migration [13].

Although our data indicate that *ISG15* over-expression is regulated by HBV infection and may trigger liver disease progression, the study has limitations. Due to the study design as a case-control study, ISG15 levels over the course of HBV infection were not assessed longitudinally and the causative effect of ISG15 levels on progression of HBV-related liver diseases could not conclusively be determined. HCC patients who donated liver tissues were in early and intermediate stages of liver cancer, which also might influence our results. Therefore, further studies in HCC patients with advanced stages of liver cancer are required to correlate *ISG15* mRNA expression with cancer progression.

In conclusion, our study shows that both the *ISG15* rs1921 variant and ISG15 overexpression are associated with HBV-related liver diseases and indicate that ISG15 may be a proviral factor and trigger progression of HBV-related liver diseases.

## MATERIALS AND METHODS

### Study design and sample collection

We randomly recruited 766 unrelated Vietnamese HBV-infected patients in a case-control design at 108 Military Central Hospital and 103 Military Hospital of the Vietnam Military Medical University, Hanoi, Vietnam, between 2012 and 2015. Patients were assigned to subgroups of disease based on clinical manifestations and liver function tests. Subgroups included chronic hepatitis B (CHB, n = 262), HBV-related liver cirrhosis (LC, n = 241) and HBV-related hepatocellular carcinoma (HCC, n = 263). Criteria for the patient classification have been described previously [49]. HBV load is quantified by qPCR and the presence of HBsAg by ELISA. Among the 263 HCC patients, 180 (68.4%) had concomitant LC and the remaining 83 patients (21.6%) did not have LC. As the healthy control (HC) group, we collected 223 blood samples from blood donors from blood bank and these healthy individuals were devoid of HBsAg, HCV and HIV infections. Neither the HCs nor HBV patients had a history of alcohol or drug use. Five ml of venous blood were collected from all participants. Serum/plasma was separated and stored at −80°C until further use. In addition, 36 dyads of liver tissue specimens (tumor and adjacent non-tumor specimens) from HCC patients undergoing surgery were collected between 2013 and 2014. HCC was confirmed histologically and classified based on the Barcelona Clinic Liver Cancer (BCLC) classification [25]. The clinical profiles of the HCC patients and data of the liver tissue specimens were shown in the Suppl. Table 1. All specimens were frozen at −80°C until use.

### Ethics statement

Informed written consent was obtained after explanation of the study at the time of sampling from all participants or from their parents if subjects were less than 18 years. The study was approved by the institutional review board of the 108 Military Central hospital and the 103 Military Hospital of the Vietnam Military Medical University, Hanoi, Vietnam.

### *ISG15* genotyping

Genomic DNA was isolated from whole blood using a DNA purification kit (Qiagen, Hilden, Germany). The *ISG15* promoter and the two exons of the gene were PCR amplified using specific sets of primers (Suppl. Table 2). PCR amplification was carried out in a 25 μl volume containing 1X PCR buffer, 0.2 mM dNTPs, 1 mM MgCl_2_, 0.15 mM of each primer, 1 unit of Taq polymerase and 50 ng of genomic DNA. Cycling conditions were denaturation at 95°C for 5 min, followed by 40 cycles of three-step cycling with denaturation (94°C, 30 sec), annealing (63°C, 35 sec), and extension (72°C, 45 sec) and final extension (72°C, 7 min). PCR products were purified by Exo-SAP-IT (USB, Affymetrix, USA) and 5 μl of products were used as sequencing templates (BigDye terminator v.1.1 cycle sequencing kit, ABI 3130XL DNA sequencer; Applied Biosystems, Foster City, USA).

### Quantification of ISG15 levels by ELISA

Soluble ISG15 serum levels were assessed in 174, 141 and 155 samples from CHB, LC and HCC patients, respectively and in 175 samples from HCs by the sandwich ISG15 ELISA kit (LifeSpan BioSciences, Eching, Germany, catalog number: MBS9302876-96). The lower detection limit was 0.5 ng/ml.

### *ISG15* mRNA expression

Total RNA was extracted from 36 dyads of liver biopsy tissues with Trizol reagent (Life Technologies, California, USA). RNA was transcribed into cDNA (QuantiTect Reverse Transcription Kit; Qiagen, Hilden, Germany). Quantification of cDNA was performed by qRT-PCR using SYBR Green PCR mix (Bioline, Luckenwalde, Germany). All reactions were performed in duplicate (LightCycler^®^480 real-time PCR system; Roche, Basel, Switzerland). The glyceraldehyde 3-phosphate dehydrogenase (*GAPDH)* gene was used as the reference gene. The specific primers used for evaluating the relative expression of *ISG15* mRNA were presented in Suppl. Table 2. Thermal cycling conditions were 2 min (95°C) followed by 45 cycles of (95°C, 5 sec), annealing (58°C, 10 sec) and extension (72°C, 20 sec). Reaction specificity was confirmed by melting curve and electrophoresis analyses. Calculation of normalized gene expression was based on the ΔΔCT method. The fold change in *ISG15* mRNA was normalized against the expressed *GAPDH* reference gene and adjusted to the calibration sample [50].

### Statistical and genetic analyses

Statistical analyses were performed using R version 3.1.2 (http://www.r-project.org) and GraphPad Prism 6 (http://www.graphpad.com)Hardy-Weinberg equilibrium deviations were calculated according to the Guo & Thompson approach by using R software. We applied binary logistic regression models adjusted for age and gender to determine *ISG15* associations with HBV-related liver diseases in co-dominant, dominant and recessive models. Univariate analysis and a multivariate linear regression model for independent factors were used to correlate ISG15 serum levels with clinical parameters. Adjusted odds ratios (OR) with 95% confidence intervals (CI) were calculated. Chi-square tests were used to test for differences of categorical variables and Mann-Whitney-Wilcoxon and Kruskal-Wallis tests were applied to compare quantitative variables. Significance was set at a value of *P* < 0.05.

## SUPPLEMENTARY MATERIALS FIGURES AND TABLES


