# Supplementary material for: Interferon-stimulated gene 15 in hepatitis B-related liver diseases
Source: Oncotarget. 2016 Sep 10;7(42):67777–87. doi: 10.18632/oncotarget.11955 (PMC5356518; doi:10.18632/oncotarget.11955)
Supplement: Supplementary file 1 [file oncotarget-07-67777-s001.pdf]

# Interferon-stimulated gene 15 in hepatitis B-related liver diseases

## Supplementary Materials

### Suppl. Table 1: Characteristics of 36 HCC patients

| Characteristics | n (%) |
|---|---|
| **Age (years)** | |
| < 40 | 3/36 (8.3) |
| 40 - 60 | 27/36 (75) |
| > 60 | 6/36 (16.7) |
| **Gender** | |
| Male | 32/36 (89) |
| Female | 4/36 (11) |
| **Etiology** | |
| HBV | 17/36 (47) |
| HCV | 2/36 (6) |
| Non-HBV/HCV | 17/36 (47) |
| **BCLC staging Classification** | |
| Stage A | 25/36 (69) |
| Stage B | 11/36 (31) |
| Stage C and D | 0/ 36 (0) |
| **Clinical parameters** | **Median (Range)** |
| AFP (IU/ml) | 240 [4.6 - 300] |
| HBV-DNA | NA |
| PLT ($10^3$/ml) | 211 [153 - 461] |
| AST (IU/ml) | 52 [21 - 415] |
| ALT (IU/ml) | 66.5 [17 - 242] |
| Total Bilirubin (µmol/l) | 27.8 [8.9 - 315] |
| Direct Bilirubin (µmol/l) | 6.7 [1 - 178] |
| Prothrombin (% of standard) | 93 [75 - 125] |
| Protein (g/l) | 73 [62 - 78] |
| Allbumin (g/l) | 40 [32 - 48] |

Abbreviation: BCLC: Barcelona Clinic Liver Cancer; HCC: hepatocellular carcinoma; AFP: Alpha feto protein; PLT: platelets; AST and ALT: aspartate and alanine amino transferase; IU: international unit; NA: not applicable.

**Suppl. Table 2: Primers used for this study**

| Primer | Sequence | Application | Fragment length |
|---|---|---|---|
| *ISG15_Pr_F* | 5'- GAG GCT GAG GTG AGA GGA TC -3' | ISG15 promoter genotyping | 715 bp |
| *ISG15_Pr_R* | 5'- GAG GGA GAC GAA AAT TGG CTG -3' | | |
| *ISG15_E1_F* | 5'- CAG TGC CTT GTG TGT GGT GG -3' | ISG15 exon1 genotyping | 578 bp |
| *ISG15_E1_R* | 5'- GAT GCT GGT GGA GGC CCT TAG -3' | | |
| *ISG15_Exp_F* | 5'- GAG AGG CAG CGA ACT CAT CT -3' | ISG15 mRNA expression | 157 bp |
| *ISG15_Exp_R* | 5'- CTT CAG CTC TGA CAC CGA CA -3' | | |
| *GADPH_F* | 5'-TGC ACC ACC AAC TGC TTA GC-3' | ISG15 mRNA expression | 87 bp |
| *GADPH_R* | 5'-GGC ATG GAC TGT GGT CAT GAG-3' | | |

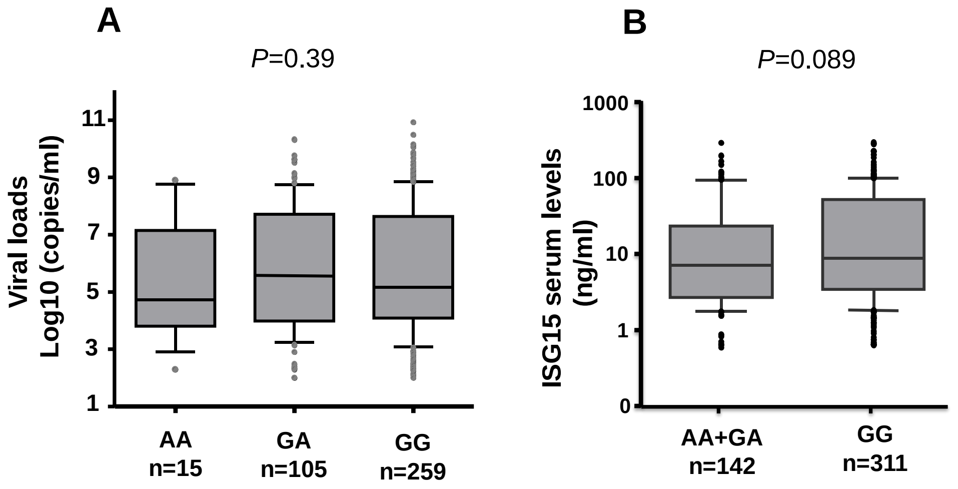


**Suppl. Figure 1: Association of viral loads and ISG15 serum levels with *ISG15* rs1921G/A variant.**

Box-plots illustrate medians with 25 and 75 percentiles with whiskers to 10 and 90 percentiles; *P* values were calculated by Krusskal-Wallis-Wilcoxon test.

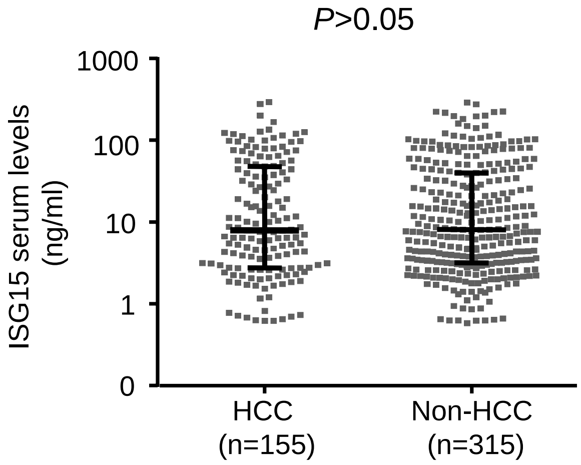


**Suppl. Figure 2: Distribution of ISG15 serum levels in HCC and non-HCC patients.**

Scatter dot plots illustrate medians with 25 and 75 percentiles with whiskers to 10 and 90 percentiles; *P* values were calculated by Krusskal-Wallis-Wilcoxon test.
